# Lithium Chloride-Induced Nuclear Envelope Remodeling in 3D Endometrial Cancer Spheroids

**DOI:** 10.3390/ijms27146352

**Published:** 2026-07-17

**Authors:** Ayhan Bilir, Berna Yıldırım, Mete Hakan Karalök, Kudret Kulak

**Affiliations:** 1 Department of Histology and Embryology, Faculty of Medicine, İstanbul Atlas University, İstanbul 34403, Turkey; berna.yildirim@atlas.edu.tr; 2 Department of Obstetrics and Gynecology, Faculty of Medicine, İstanbul Atlas University, İstanbul 34403, Turkey; hakan.karalok@atlas.edu.tr; 3 Department of Pediatrics, Faculty of Medicine, İstanbul Atlas University, İstanbul 34403, Turkey; kudret.kulak@atlas.edu.tr

**Keywords:** lithium chloride, nuclear envelope remodeling, ultrastructure, 3D spheroids, endometrial cancer, transmission electron microscopy

## Abstract

Selective remodeling of nuclear architecture under pharmacological stress remains incompletely characterized in three-dimensional cancer models, particularly at the ultrastructural level. Lithium chloride (LiCl) is widely used as a cellular stressor capable of altering membrane organization, proliferation, and organelle dynamics; however, its effects on nucleus-associated structural remodeling in three-dimensional tumor systems remain unclear. In the present study, Ishikawa endometrial carcinoma spheroids were exposed to LiCl (10 or 50 mM) for up to 96 h, and cellular responses were evaluated using cell cycle analysis, 5-bromo-2′-deoxyuridine (BrdU) incorporation, viability assays, and transmission electron microscopy. LiCl exposure was associated with sustained accumulation of cells in the G_0_/G_1_ phase and suppression of DNA synthesis without evidence of a dominant apoptotic or necrotic response under the examined conditions. Ultrastructural analyses showed prominent nuclear envelope elongation, membrane reorganization, and perinuclear double-membraned structures, particularly under prolonged LiCl exposure. These alterations were observed alongside mitochondrial and endomembrane remodeling, supporting the presence of a broader stress-associated ultrastructural adaptation response within LiCl-treated spheroids. Importantly, the present study is morphology-focused and descriptive in scope. Although some ultrastructural features were morphology-compatible with nucleophagy-associated remodeling, no molecular validation using LC3, p62/SQSTM1, LAMP1/lysosomal colocalization, lamin degradation, or autophagic-flux assays was performed. Therefore, the findings should be interpreted as evidence of stress-associated nuclear envelope remodeling rather than mechanistic proof of nucleophagy. Collectively, this study identifies nucleus-associated ultrastructural remodeling as a prominent cellular response to LiCl-induced stress in 3D endometrial cancer spheroids and highlights the utility of three-dimensional tumor models for investigating stress-related architectural adaptations in cancer cells.

## 1. Introduction

Autophagy is a fundamental intracellular degradation pathway that enables cells to adapt to metabolic imbalance, oxidative stress, and organelle dysfunction through the lysosomal turnover of intracellular components [1]. Initially regarded as a bulk degradative mechanism, autophagy is now recognized as a selective and context-dependent process capable of targeting specific cellular structures in response to defined stress conditions [2,3]. In addition to cytoplasmic organelles, increasing evidence suggests that stress-associated remodeling may also involve nuclear components and nuclear membrane dynamics under certain pathological conditions [2,3,4].

The selective turnover or remodeling of nuclear-associated structures, often discussed within the framework of nucleophagy, has been extensively characterized in yeast and lower eukaryotes; however, its structural and biological relevance in mammalian cancer systems remains incompletely understood [4,5,6,7,8,9,10,11]. In mammalian cells, nucleus-associated autophagic responses have largely been inferred from molecular observations rather than direct ultrastructural evidence [3,12,13,14]. Furthermore, distinguishing stress-associated nuclear remodeling from classical nuclear degeneration or other membrane-associated alterations remains challenging when interpretation is based primarily on morphology [4,5,6,15,16]. Consequently, ultrastructural analyses remain important for characterizing nucleus-associated membrane dynamics under cellular stress conditions.

Autophagic pathways are coordinated by a conserved set of autophagy-related (ATG) proteins, among which the ATG8/LC3 family plays a central role in membrane dynamics and cargo recognition [17]. LC3 participates in autophagosome membrane elongation and mediates selective autophagy through interactions with adaptor proteins such as p62 and NBR1 [15,16,17]. Although LC3 has been implicated in nuclear-associated autophagic events, its presence alone is insufficient to establish nucleophagy without complementary structural or functional validation.

Lithium chloride (LiCl) was selected as an experimental stressor in the present study because of its well-documented effects on intracellular signaling, cellular proliferation, and stress-associated remodeling pathways [18]. LiCl exerts pleiotropic biological actions, including inhibition of glycogen synthase kinase-3β (GSK-3β), modulation of inositol metabolism, and alteration of IP_3_- and mTOR-associated signaling pathways, all of which may influence cellular growth, membrane organization, and stress adaptation [18,19]. In endometrial carcinoma and other malignancies, LiCl exposure has been associated with cytostatic responses accompanied by cell cycle arrest, altered proliferative activity, and organelle-associated structural remodeling [20,21].

In addition to its antiproliferative effects, LiCl has also been reported to influence autophagy-associated pathways and intracellular membrane dynamics [18,19]. Previous studies have demonstrated that LiCl promotes selective autophagy, particularly mitophagy, in various experimental systems, and emerging evidence suggests that lithium exposure may also affect nuclear-associated stress responses [18,22]. Moreover, LiCl exposure has been associated with broader ultrastructural changes involving membrane reorganization, endoplasmic reticulum alterations, and perturbations in organelle architecture across multiple experimental systems [23,24,25,26,27,28,29,30,31,32]. However, whether LiCl similarly induces stress-associated remodeling of the nuclear compartment in mammalian cancer spheroids remains insufficiently characterized.

Recent ultrastructural studies, including previous studies using three-dimensional (3D) endometrial cancer spheroids, have documented LiCl-induced mitochondrial remodeling and mitophagy-like features linked to mitochondrial stress and endoplasmic reticulum-associated autophagic responses [18,33,34]. However, whether LiCl similarly provokes nucleus-directed autophagic remodeling in mammalian cancer cells has not been systematically examined.

The present study addresses this question through a detailed ultrastructural analysis of LiCl-treated Ishikawa endometrial carcinoma spheroids cultured in a three-dimensional context. In parallel with ultrastructural evaluation, cell cycle distribution, DNA synthesis, and cell viability were examined to characterize the broader cellular stress response associated with LiCl exposure. Ultrastructural analyses showed nuclear envelope elongation, membrane reorganization, and perinuclear double-membraned structures under LiCl exposure. These alterations occurred in parallel with proliferative suppression and G_0_/G_1_-associated cell cycle accumulation in the absence of a dominant apoptotic response. However, because no LC3, p62/SQSTM1, LAMP1/lysosomal colocalization, lamin degradation, or autophagic-flux analyses were performed, the observed structures cannot be interpreted as mechanistic evidence of nucleophagy. Rather, they should be considered morphology-compatible, stress-associated nuclear envelope remodeling events. Collectively, this study describes a reproducible ultrastructural phenotype associated with LiCl-induced stress in 3D endometrial cancer spheroids and highlights the utility of three-dimensional tumor models for investigating stress-associated architectural adaptation in cancer cells.

## 2. Results

### 2.1. LiCl Exposure Is Associated with Proliferative Suppression and G_0_/G_1_-Associated Cell Cycle Redistribution in 3D Ishikawa Spheroids

**Figure 1:**
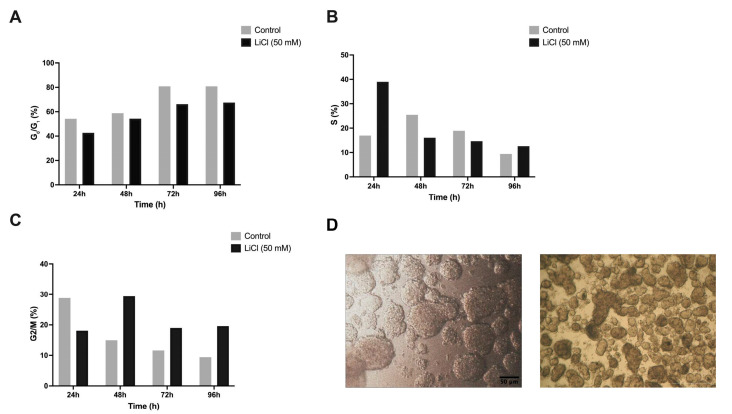
Effects of high-dose LiCl (50 mM) on cell cycle distribution and representative spheroid morphology in 3D Ishikawa spheroids. (**A**–**C**) Percentage distribution of cells in G_0_/G_1_, S, and G_2_/M phases at 24, 48, 72, and 96 h under control and LiCl-treated conditions, determined by propidium iodide staining and flow cytometric analysis. The panels are presented as descriptive cell cycle profiles. Because this figure is presented without error bars or statistical significance indicators, these data should be interpreted as treatment- and time-associated descriptive trends. (**D**) Representative bright-field images of control and LiCl-treated spheroids at 48 h, included as morphological context and not as part of the flow cytometric cell cycle quantification. LiCl-treated spheroids appeared more compact and exhibited reduced peripheral cell dispersion compared with controls. Scale bars: 50 µm.

To evaluate the effects of lithium chloride (LiCl) on proliferative activity, cell cycle distribution was analyzed in 3D Ishikawa spheroids following 24, 48, 72, and 96 h of exposure. As shown in Figure 1A–C, LiCl treatment altered the relative distribution of cells across the G_0_/G_1_, S, and G_2_/M phases over time. LiCl-treated spheroids exhibited a relative enrichment of the G_0_/G_1_-associated population, particularly at 72 and 96 h, accompanied by a reduction in the S-phase fraction following an initial increase at 24 h. Alterations in the G_2_/M-associated population were also observed, indicating a broader redistribution of cell cycle phases under LiCl exposure. Because Figure 1 is presented descriptively without error bars or statistical significance indicators, these cell cycle changes should be interpreted as descriptive treatment- and time-associated trends rather than definitive quantitative comparisons. The representative bright-field images in Figure 1D are provided separately as morphological context and were not used for cell cycle quantification.

### 2.2. LiCl Treatment Induces Sustained Suppression of DNA Synthesis in 3D Ishikawa Spheroids

**Figure 2:**
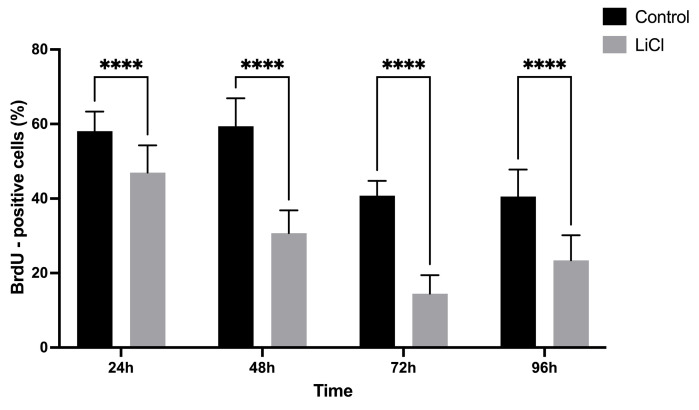
Effect of high-dose LiCl (50 mM) on BrdU incorporation in 3D Ishikawa spheroids. Quantification of BrdU-positive cells in control and LiCl-treated spheroids at 24, 48, 72, and 96 h was determined by immunostaining. BrdU-positive nuclei were identified based on nuclear DAB staining intensity. Data are presented as mean ± SD derived from independent experiments (*n* ≥ 3). Statistical analysis was performed using two-way ANOVA followed by Sidak’s multiple comparisons test; **** *p* < 0.0001 versus corresponding time-matched control.

Cellular proliferation was further evaluated using bromodeoxyuridine (BrdU) incorporation analysis. As shown in Figure 2, LiCl-treated spheroids showed a marked reduction in the proportion of BrdU-positive cells compared with corresponding controls at all examined time points. This decrease was consistently observed at 24, 48, 72, and 96 h, indicating sustained suppression of DNA synthesis under LiCl treatment conditions. Two-way ANOVA analysis confirmed a treatment-associated effect of LiCl on BrdU incorporation across time points.

### 2.3. LiCl-Associated Reduction in Viable Cell Fraction Occurs Without a Dominant Apoptotic Response

**Figure 3:**
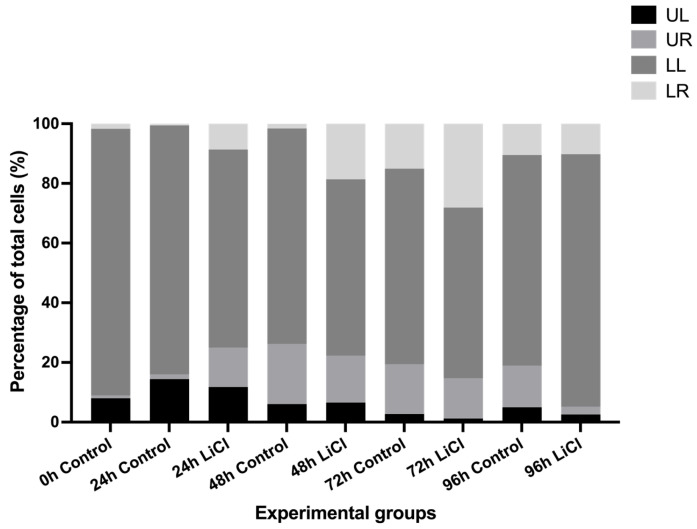
Flow cytometric analysis of viable, apoptotic, and necrotic cell populations in LiCl-treated Ishikawa 3D spheroids. Annexin V–FITC/propidium iodide staining was used to classify gated intact cells as viable cells (Annexin V^−^/PI^−^), early apoptotic cells (Annexin V^+^/PI^−^), late apoptotic cells (Annexin V^+^/PI^+^), or necrotic cells (Annexin V^−^/PI^+^). Data are presented as percentages of gated events across independent experiments (*n* ≥ 3). Gating was established using unstained and single-stained controls. Small debris and poorly dissociated events were excluded from analysis; therefore, reduced viable fractions should not be interpreted as total cell loss or as proportional redistribution into apoptotic or necrotic populations.

To determine whether LiCl-associated alterations in spheroid viability were accompanied by apoptotic or necrotic cell death, Annexin V–FITC/propidium iodide (PI) staining was performed (Figure 3). Flow cytometric analysis revealed a reduction in the proportion of viable gated cells (Annexin V^−^/PI^−^) in LiCl-treated spheroids compared with corresponding controls across the examined time points (Figure 3). However, this reduction was not accompanied by a proportional increase in early apoptotic (Annexin V^+^/PI^−^), late apoptotic (Annexin V^+^/PI^+^), or necrotic (Annexin V^−^/PI^+^) populations. Instead, these subpopulations remained comparatively limited throughout the experimental period.

This apparent discrepancy may reflect the fact that Annexin V/PI quadrant analysis reports the relative distribution of gated intact events rather than total cell recovery from spheroids. Small debris, fragmented material, and poorly dissociated events were excluded during gating and therefore were not represented within the viable, apoptotic, or necrotic quadrants. In addition, total cell recovery after spheroid dissociation was not quantified. Thus, the reduced viable fraction should be interpreted as evidence of altered viable gated-cell distribution rather than as a direct measure of proportional conversion into classical apoptotic or necrotic populations.

### 2.4. LiCl Induces Stress-Associated Ultrastructural Remodeling Localized Predominantly to the Nuclear Compartment

**Figure 4:**
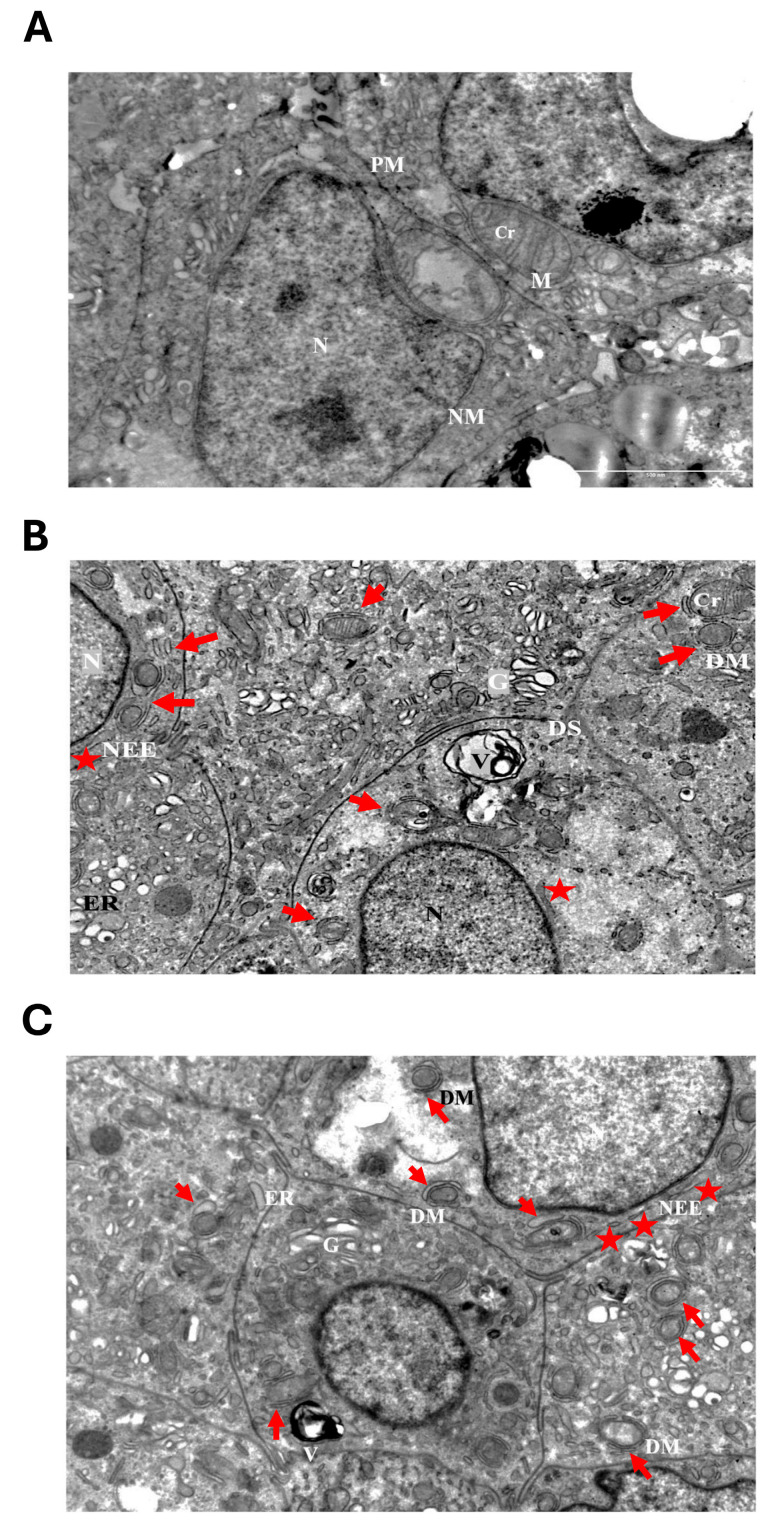
Representative ultrastructural features of stress-associated nuclear envelope remodeling in LiCl-treated Ishikawa spheroids. (**A**) Control spheroids at 72 h displaying intact plasma membrane (PM), continuous nuclear membrane/nuclear envelope (NM/NE), and preserved mitochondrial structure with intact cristae. (**B**) Spheroids treated with low-dose LiCl (10 mM) showing early nuclear envelope elongation (NEE; asterisks) and membrane-associated vesicular structures. (**C**) Spheroids treated with high-dose LiCl (50 mM) showing more extensive membrane remodeling, including double-membraned vesicular structures (DM; arrows) localized near the nuclear periphery and associated with nuclear envelope deformation. Images are representative of reproducible qualitative ultrastructural patterns. Formal morphometric quantification was not performed. Scale bars: 500 nm. Abbreviations: PM, plasma membrane; Cr, cristae; M, mitochondrion; N, nucleus; NM, nuclear membrane; NEE, nuclear envelope elongation; ER, endoplasmic reticulum; G, Golgi apparatus; DS, desmosome; V, vesicle; DM, double membrane.

**Figure 5:**
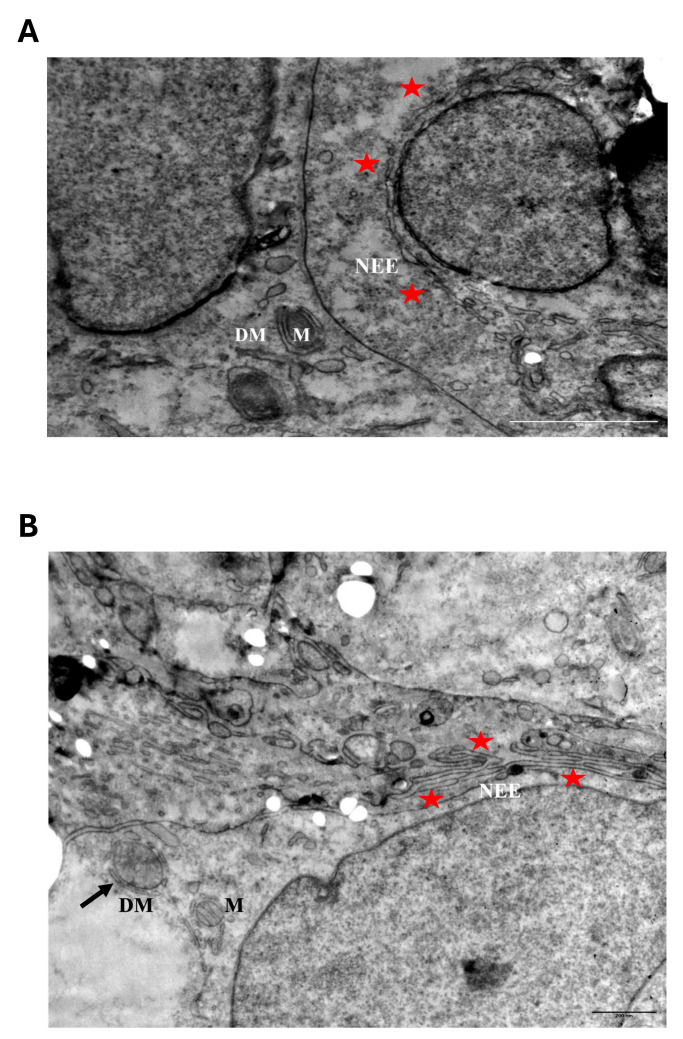
Representative high-magnification views of stress-associated nuclear envelope remodeling in LiCl-treated spheroids (50 mM). (**A**) High-magnification view showing nuclear envelope elongation (NEE; asterisks) and associated membrane rearrangements at the nuclear periphery. (**B**) Double-membraned vesicular structures (DM; arrows) and nuclear envelope deformation in close proximity to mitochondrial structures. These features are morphology-compatible with nucleus-associated membrane remodeling but do not establish nucleophagy in the absence of molecular autophagy or lysosomal validation. Scale bars: 500 nm. Abbreviations: NEE, nuclear envelope elongation; DM, double membrane; M, mitochondrion.

Transmission electron microscopy showed reproducible qualitative ultrastructural alterations in LiCl-treated spheroids, with prominent changes localized to the nuclear compartment (Figure 4 and Figure 5). Control spheroids exhibited relatively preserved nuclear morphology, intact plasma membranes, and organized intracellular architecture (Figure 4A). In contrast, LiCl-treated spheroids displayed nuclear envelope elongation, irregular nuclear contours, membrane reorganization, and perinuclear double-membraned structures, particularly after prolonged exposure to 50 mM LiCl (Figure 4B,C and Figure 5A,B).

In addition to nuclear-associated alterations, LiCl-treated spheroids also showed mitochondrial remodeling, cytoplasmic vacuolization, dilated endoplasmic reticulum cisternae, and membrane-associated vesicular structures. However, many of the observed remodeling events were localized at or around the nuclear envelope, suggesting that the nuclear compartment represents a prominent site of stress-associated ultrastructural adaptation under sustained LiCl exposure. Notably, these structural alterations were observed in parallel with reduced BrdU incorporation and G_0_/G_1_-associated cell cycle redistribution. However, these findings remain correlative, and the present data do not establish a causal link between cell-cycle changes and nuclear envelope remodeling.

## 3. Discussion

The present study shows that lithium chloride (LiCl) exposure in three-dimensional (3D) Ishikawa endometrial cancer spheroids is associated with redistribution of cells toward the G_0_/G_1_ phase together with sustained suppression of DNA synthesis, indicating a predominantly cytostatic response under the examined conditions. Similar lithium-associated antiproliferative effects have previously been reported in multiple cancer models and have been linked to disruption of signaling pathways involved in cell cycle regulation, including GSK-3β- and inositol-dependent mechanisms [23,24]. Importantly, the increase in the G_0_/G_1_-associated population observed in the present study was not accompanied by accumulation of sub-G_1_ fractions or other cytometric features typically associated with apoptotic progression. These findings support the interpretation that LiCl primarily interferes with proliferative signaling and cellular growth dynamics rather than inducing acute apoptotic cytotoxicity. Previous studies have also shown that LiCl-mediated modulation of GSK-3β and inositol/IP_3_-associated pathways may influence membrane organization, proliferation, and stress-associated cellular remodeling [18,19,23,24]. Although these molecular mechanisms were not directly investigated in the present study, they have previously been associated with stress-related membrane remodeling responses observed in LiCl-treated spheroids.

Consistent with this interpretation, Annexin V–FITC/propidium iodide analysis showed a reduction in the viable gated-cell fraction following LiCl exposure without a corresponding proportional increase in early apoptotic, late apoptotic, or necrotic populations. This pattern should be interpreted with caution because Annexin V/PI analysis was performed on gated intact events after spheroid dissociation. Debris, small fragmented material, and poorly dissociated events were excluded from quadrant analysis, and total cell recovery was not quantified. Therefore, the reduced viable fraction may reflect a combination of altered gated-cell distribution, dissociation-related recovery differences, and non-classical stress-associated impairment rather than direct conversion of viable cells into classical apoptotic or necrotic populations. Similar patterns of lithium-associated growth suppression, metabolic stress, or functional impairment in the absence of pronounced caspase-dependent apoptotic execution have been reported previously [33,35,36]. Accordingly, the present findings support the presence of a non-canonical stress response characterized by proliferative arrest and structural remodeling rather than a dominant regulated cell death phenotype [37]. The use of a 3D spheroid system is particularly relevant in this context because it preserves tissue-like architecture, spatial organization, nutrient gradients, and cell–cell interactions that are not fully reproduced in conventional monolayer cultures [38]. These features may contribute to heterogeneous stress responses within the spheroid structure and likely influence both the functional and ultrastructural alterations observed following LiCl exposure. Although region-specific analyses, such as comparisons between spheroid core and peripheral regions, were not performed in the present study, future spatially resolved imaging and three-dimensional reconstruction approaches may help clarify localization-dependent stress responses within the spheroid microenvironment.

Transmission electron microscopy showed that LiCl exposure was associated with prominent qualitative ultrastructural alterations, with substantial remodeling localized to the nuclear compartment. Lithium-treated spheroids exhibited nuclear envelope elongation, irregular nuclear contours, and perinuclear double-membraned structures in the absence of classical ultrastructural hallmarks of apoptosis. These alterations are consistent with stress-associated membrane remodeling involving the nuclear compartment and are morphology-compatible with previously described nucleus-associated autophagic phenotypes reported in yeast and mammalian systems [2,3]. However, morphology alone is insufficient to establish nucleophagy. In the absence of LC3, p62/SQSTM1, LAMP1/lysosomal colocalization, lamin degradation, or autophagic-flux assays, the observed structures should not be interpreted as mechanistic evidence of nucleophagy.

Similar membrane configurations may also arise during endoplasmic reticulum- or Golgi-associated membrane remodeling under conditions of sustained cellular stress. As previously reported, structures morphologically compatible with mitophagy-like processes were also observed, consistent with earlier descriptions of lithium-associated mitochondrial stress and membrane remodeling [18,23,39]. Nevertheless, many of the membrane-associated alterations identified in the present study were localized at or around the nuclear envelope, indicating structural involvement of the nuclear compartment during sustained LiCl-associated stress exposure.

The preferential localization of these alterations at the nuclear periphery further supports the interpretation of a nucleus-associated remodeling response rather than a purely cytoplasmic process. Formal morphometric quantification of TEM findings was not performed. Therefore, the present study does not provide numerical measurements of nuclear envelope elongation frequency, the number of double-membraned structures per cell or per nuclear perimeter, the percentage of affected spheroid cells, or blinded scoring across biological replicates. These limitations should be addressed in future investigations designed to evaluate the spatial distribution, frequency, and three-dimensional specificity of LiCl-associated nuclear envelope remodeling.

The observed nuclear alterations appeared more prominent under prolonged high-dose LiCl exposure. Lower LiCl concentrations (10 mM) were associated with comparatively subtle and early structural changes, whereas prolonged exposure to 50 mM LiCl resulted in more extensive nuclear envelope remodeling. This pattern suggests that sustained pharmacological stress may progressively alter nuclear membrane organization and supports the interpretation that the nucleus constitutes a major site of stress-associated ultrastructural adaptation under prolonged LiCl exposure. However, the interpretation of these dose-associated findings should consider the high concentration of LiCl used in the main experimental condition. Although 50 mM LiCl was selected as a pharmacological stress condition based on previous in vitro studies, this concentration may induce broad osmotic or non-specific stress responses. Because osmolarity-matched controls and additional lower-dose functional validations were not included, LiCl-specific effects cannot be fully separated from general hyperosmotic or cytotoxic stress in the present study.

Notably, nuclear remodeling occurred in parallel with G_0_/G_1_-associated cell cycle redistribution and suppression of DNA synthesis. However, this relationship remains correlative. Because no experiments were performed to directly manipulate cell-cycle arrest independently of LiCl exposure, no causal relationship between G_0_/G_1_ accumulation, reduced BrdU incorporation, and nuclear envelope remodeling can be established based on the current findings. From a broader cancer biology perspective, these observations suggest that nucleus-associated stress responses may contribute to cellular adaptation during pharmacological stress, particularly within three-dimensional tumor systems that better preserve tissue-like organization. Additional limitations of the present study include the absence of spatially resolved analysis within spheroid regions, the lack of formal TEM morphometric quantification, and the use of a single endometrial cancer cell line. The use of only Ishikawa spheroids limits the generalizability of the findings to other molecular or histological subtypes of endometrial cancer. Future studies should validate the key ultrastructural and functional observations in additional endometrial cancer models and integrate molecular and spatially resolved imaging approaches to further characterize the biological significance and mechanistic basis of LiCl-associated nuclear envelope remodeling. Although the observed nuclear envelope elongation and double-membraned structures are morphology-compatible with previously described nucleus-associated autophagic phenotypes, no direct molecular validation of autophagy-related pathways was performed in the present study. Specifically, LC3 recruitment, p62/SQSTM1 involvement, LAMP1 or lysosomal colocalization, lamin degradation, and autophagic-flux assays were not evaluated. Therefore, these findings should be interpreted as morphology-based and hypothesis-generating rather than definitive evidence of nucleophagy. Future investigations incorporating molecular imaging, functional autophagy assays, lysosomal colocalization studies, lamin-related analyses, and higher-resolution spatial approaches will be required to determine the molecular identity and mechanistic significance of the observed nuclear remodeling events.

## 4. Materials and Methods

### 4.1. Three-Dimensional Spheroid Culture and Lithium Chloride Exposure

Three-dimensional tumor spheroids were generated using a liquid-overlay (agar-based) culture method. Ishikawa human endometrial adenocarcinoma cells (ATCC, RRID: CVCL_2529) were maintained in DMEM/F12 medium (Gibco, Thermo Fisher Scientific, Waltham, MA, USA) supplemented with 10% fetal bovine serum (FBS; Sigma-Aldrich, St. Louis, MO, USA), 1% L-glutamine, and 1% penicillin–streptomycin under standard humidified culture conditions (37 °C, 5% CO_2_). For spheroid formation, single-cell suspensions were seeded at a density of 3 × 10^5^ cells/mL in ultra-low-attachment six-well plates (Corning Inc., Corning, NY, USA) pre-coated with 3% Noble agar (Sigma-Aldrich, St. Louis, MO, USA). Compact multicellular spheroids formed within 48 h under these conditions.

Spheroids within a diameter range of approximately 120–300 μm were selected for experimental treatment to reduce variability related to spheroid size. Lithium chloride (LiCl; Sigma-Aldrich, St. Louis, MO, USA) was freshly prepared in sterile distilled water and added to culture medium at a final concentration of 50 mM for 24, 48, 72, or 96 h. Control spheroids received an equivalent volume of vehicle alone. To evaluate early ultrastructural alterations, additional experiments were performed using a lower LiCl concentration of 10 mM, whereas quantitative functional analyses primarily focused on the 50 mM treatment condition [39,40].

The selected LiCl concentrations were based on previous in vitro studies in which high-dose LiCl was used as a pharmacological stress condition to induce measurable changes in proliferation, organelle architecture, and ultrastructural remodeling. The 50 mM condition was therefore used as a high-intensity stress model rather than as a clinically equivalent exposure. The lower 10 mM concentration was included to evaluate whether early ultrastructural alterations could also be observed under a less intense LiCl exposure. Nevertheless, because osmolarity-matched controls were not included, the possibility that part of the observed response reflects hyperosmotic or non-specific cytotoxic stress cannot be excluded.

Bright-field evaluation was used to monitor gross spheroid morphology, including compaction and peripheral cell dispersion, during the treatment period. However, systematic quantitative measurements of spheroid diameter, compaction index, or dissociation efficiency were not performed at each time point. Therefore, possible effects of LiCl on spheroid size distribution and dissociation-related cell recovery are acknowledged as methodological limitations. The use of 3D spheroid cultures enabled preservation of cell–cell interactions and diffusion gradients, providing a physiologically relevant tumor microenvironment compared with conventional monolayer cultures.

### 4.2. Bromodeoxyuridine Incorporation Assay

DNA synthesis was evaluated using 5-bromo-2′-deoxyuridine (BrdU) incorporation analysis. BrdU (20 μM; Sigma-Aldrich, St. Louis, MO, USA) was added directly to spheroid cultures during the final 4 h of treatment. Following exposure, spheroids were fixed in 4% paraformaldehyde (Electron Microscopy Sciences, Hatfield, PA, USA), embedded in paraffin, and sectioned at 5 μm thickness. After deparaffinization and rehydration, antigen retrieval was performed using citrate buffer (pH 6.0), followed by DNA denaturation with 2 N HCl. Sections were incubated with a mouse anti-BrdU primary antibody (Lab Vision, Thermo Fisher Scientific, Fremont, CA, USA), followed by biotinylated secondary antibody and streptavidin–peroxidase conjugate (Lab Vision, Thermo Fisher Scientific, Fremont, CA, USA). Immunoreactivity was visualized using diaminobenzidine (DAB; Lab Vision, Thermo Fisher Scientific, Fremont, CA, USA), and sections were counterstained with hematoxylin. BrdU-positive nuclei were quantified in five randomly selected microscopic fields per section by two independent observers blinded to treatment conditions. Images were acquired using bright-field microscopy under identical acquisition settings. Results were expressed as the percentage of BrdU-positive nuclei relative to the total number of nuclei evaluated [40].

### 4.3. Flow Cytometric Cell Cycle Analysis

For cell cycle analysis, spheroids were dissociated into single-cell suspensions using Accutase (Sigma-Aldrich, St. Louis, MO, USA). Cells were fixed in cold 70% ethanol and stored at –20 °C overnight. Prior to analysis, samples were treated with RNase A (Sigma-Aldrich) and stained with propidium iodide (PI; Sigma-Aldrich) for nuclear DNA labeling. Flow cytometric acquisition was performed using a BD FACSCalibur system (BD Biosciences, San Jose, CA, USA). DNA content distributions were analyzed using ModFit LT software version 3.0 (Verity Software House, Topsham, ME, USA) to estimate the relative proportions of cells in the G_0_/G_1_, S, and G_2_/M phases. Each experimental condition was analyzed in triplicate.

### 4.4. Annexin V–FITC/Propidium Iodide Assay

Cell viability and apoptotic responses were evaluated using an Annexin V–FITC/propidium iodide (PI) apoptosis detection kit (BD Biosciences, San Jose, CA, USA) according to the manufacturer’s instructions. Dissociated cells were incubated with Annexin V–FITC and PI in binding buffer under light-protected conditions. Data acquisition was performed using a BD FACSCalibur flow cytometer (BD Biosciences, San Jose, CA, USA), and at least 10,000 events were collected per sample. Cell populations were categorized as viable, early apoptotic, late apoptotic, or necrotic according to Annexin V–FITC and PI signal distribution. Gating was established using unstained and single-stained controls, and analysis was restricted to the intact-cell gate based on forward- and side-scatter characteristics. Small debris and poorly dissociated events were excluded from quadrant analysis. Because total cell recovery after spheroid dissociation was not quantitatively measured, decreases in the viable fraction should not be interpreted as being fully redistributed into Annexin V-positive or PI-positive quadrants. This assay was used to assess apoptotic responses and was not intended to define autophagy-related cell death mechanisms.

### 4.5. Transmission Electron Microscopy (TEM)

Ultrastructural analysis was performed using transmission electron microscopy (TEM). Spheroids were fixed in 2.5% glutaraldehyde (Electron Microscopy Sciences, Hatfield, PA, USA) prepared in 0.1 M sodium cacodylate buffer for 2 h at room temperature, followed by post-fixation in 1% osmium tetroxide osmium tetroxide (Electron Microscopy Sciences, Hatfield, PA, USA) for 1 h. Samples were contrasted with 2% aqueous uranyl acetate (Sigma-Aldrich, St. Louis, MO, USA) prior to dehydration through graded acetone series and embedding in epoxy resin (Electron Microscopy Sciences, Hatfield, PA, USA). Ultrathin sections were prepared using an ultramicrotome (LKB 2088 Ultrotome V), mounted on copper grids, and counterstained with uranyl acetate and lead citrate. Imaging was conducted using a JEOL JEM-1400 Plus transmission electron microscope (JEOL Ltd., Tokyo, Japan) operating at 80 kV. Representative micrographs were selected from reproducible ultrastructural patterns observed across multiple spheroids and independent imaging sessions. TEM analysis was performed as a qualitative and descriptive ultrastructural assessment. Nuclear envelope elongation, perinuclear double-membraned structures, and membrane-associated remodeling were evaluated based on reproducible morphological patterns observed in representative sections. Formal morphometric quantification, including the frequency of nuclear envelope elongation, number of double-membraned structures per cell or per nuclear perimeter, percentage of affected spheroid cells, and blinded scoring across biological replicates, was not performed in the present study.

### 4.6. Statistical Analysis

All experiments were performed using three independent biological replicates. Quantitative data are expressed as mean ± standard deviation. Statistical analyses were performed using GraphPad Prism software (version 10.2.1, GraphPad Software, San Diego, CA, USA). Differences between experimental groups were analyzed using two-way ANOVA followed by Tukey’s multiple comparisons test for cell cycle analysis and Sidak’s multiple comparisons test for BrdU incorporation and Annexin V assays. Post hoc comparisons were performed between corresponding control and LiCl-treated groups at each time point. Statistical significance was defined as *p* < 0.05.

## 5. Conclusions

This study shows that lithium chloride exposure is associated with nuclear envelope remodeling in three-dimensional Ishikawa endometrial cancer spheroids, occurring alongside G_0_/G_1_-associated cell cycle redistribution and reduced DNA synthesis in the absence of a dominant classical apoptotic response. Ultrastructural analysis revealed nuclear envelope elongation, membrane reorganization, and perinuclear double-membraned structures, particularly under prolonged high-dose LiCl exposure.

These findings should be interpreted cautiously. Although the observed structures are morphology-compatible with nucleophagy-associated remodeling, the absence of LC3, p62/SQSTM1, LAMP1/lysosomal colocalization, lamin degradation, and autophagic-flux assays prevents mechanistic confirmation of nucleophagy. In addition, the use of a high LiCl concentration, lack of osmolarity-matched controls, absence of formal TEM quantification, and use of a single endometrial cancer cell line limit the mechanistic and generalizable interpretation of the findings.

Collectively, this study identifies stress-associated nuclear envelope remodeling as a prominent morphology-based response to LiCl exposure in 3D endometrial cancer spheroids. The findings provide a descriptive framework for future studies designed to test whether these nuclear alterations represent bona fide nucleophagy, broader stress-associated membrane remodeling, or a combination of LiCl-specific and non-specific osmotic stress responses.

## Data Availability

The data supporting the findings of this study are included in the manuscript and its figures. Additional data are available from the corresponding author upon reasonable request.

## Abbreviations

The following abbreviations are used in this manuscript:

3D	Three-dimensional
LiCl	Lithium chloride
TEM	Transmission electron microscopy
BrdU	Bromodeoxyuridine
G_0_/G_1_	Gap 0/Gap 1 phase
S phase	DNA synthesis phase
G_2_/M	Gap 2/Mitosis phase
PI	Propidium iodide
DM	Double membrane
NEE	Nuclear envelope elongation
ER	Endoplasmic reticulum
G	Golgi apparatus
DS	Desmosome
Cr	Cristae
PM	Plasma membrane
NE/NM	Nuclear envelope/nuclear membrane
N	Nucleus
M	Mitochondrion
